# Repeated Evolution of Inactive Pseudonucleases in a Fungal Branch of the Dis3/RNase II Family of Nucleases

**DOI:** 10.1093/molbev/msaa324

**Published:** 2020-12-12

**Authors:** Elizabeth R Ballou, Atlanta G Cook, Edward W J Wallace

**Affiliations:** 1 Institute for Microbiology and Infection, School of Biosciences, University of Birmingham, Birmingham, United Kingdom; 2 Wellcome Centre for Cell Biology, School of Biological Sciences, University of Edinburgh, Edinburgh, United Kingdom; 3 Institute for Cell Biology and SynthSys, School of Biological Sciences, University of Edinburgh, Edinburgh, United Kingdom

**Keywords:** pseudoenzyme, protein evolution, RNA-binding protein, nuclease, fungi

## Abstract

The RNase II family of 3′–5′ exoribonucleases is present in all domains of life, and eukaryotic family members Dis3 and Dis3L2 play essential roles in RNA degradation. Ascomycete yeasts contain both Dis3 and inactive RNase II-like “pseudonucleases.” The latter function as RNA-binding proteins that affect cell growth, cytokinesis, and fungal pathogenicity. However, the evolutionary origins of these pseudonucleases are unknown: What sequence of events led to their novel function, and when did these events occur? Here, we show how RNase II pseudonuclease homologs, including *Saccharomyces cerevisiae* Ssd1, are descended from active Dis3L2 enzymes. During fungal evolution, active site mutations in Dis3L2 homologs have arisen at least four times, in some cases following gene duplication. In contrast, N-terminal cold-shock domains and regulatory features are conserved across diverse dikarya and mucoromycota, suggesting that the nonnuclease function requires these regions. In the basidiomycete pathogenic yeast *Cryptococcus neoformans*, the single Ssd1/Dis3L2 homolog is required for cytokinesis from polyploid “titan” growth stages. This phenotype of *C. neoformans* Ssd1/Dis3L2 deletion is consistent with those of inactive fungal pseudonucleases, yet the protein retains an active site sequence signature. We propose that a nuclease-independent function for Dis3L2 arose in an ancestral hyphae-forming fungus. This second function has been conserved across hundreds of millions of years, whereas the RNase activity was lost repeatedly in independent lineages.

## Introduction

Protein function evolves such that some descendants of an enzyme become “pseudoenzymes” with conserved structure but no catalytic activity (Murphy et al. 2017; Ribeiro et al. 2019). Distinct families of RNase enzymes regulate gene expression by catalytically degrading RNA (Houseley and Tollervey 2009), as part of a wider set of RNA-binding proteins (RBPs) that regulate all stages of the mRNA life cycle (Singh et al. 2015). Some functional RBPs are pseudonucleases, where inactivation of the nuclease active site was accompanied by, or preceded by, gain-of-function in other domains (Glavan et al. 2006; Chen et al. 2015; Lazzaretti et al. 2016; Yang et al. 2016). How could such differences in function have evolved? One possibility is that, first, the ability of a nuclease to bind RNA substrates was enhanced in other domains, as a secondary “moonlighting” function. Subsequently, the ancestral enzymatic activity was lost whereas the moonlighting activity was retained (Jeffery 2019). Understanding this order of events can help identify conserved activities underlying pleiotropic phenotypes.

### RNase II/Dis3 Family Exoribonucleases

Members of the RNase II/Dis3 family of 3′–5′ exoribonucleases play important roles across the tree of life, including the founding member of the family, *Escherichia coli* RNase II. In eukaryotes, RNase II homologs include Dis3 and Dis3-like proteins. Dis3 is the essential nuclease component of the eukaryotic RNA exosome, a large protein complex that is responsible for bulk RNA turnover and processing (Dos Santos et al. 2018). In animals, Dis3 and the paralogous Dis3-like protein (Dis3L1) are present in nuclear and cytoplasmic exosomes, respectively (Staals et al. 2010; Tomecki et al. 2010). Another homolog, Dis3-like 2 (Dis3L2) specifically degrades poly(U)-tailed mRNAs, such as products of the terminal-U-transferases (Malecki et al. 2013), in *Schizosaccharomyces pombe*. This role is conserved in mammalian Dis3L2 (Ustianenko et al. 2013). A more distant homolog, Dss1, is the active subunit of the mitochondrial degradosome in fungi (Razew et al. 2018).

**Fig. 1.**
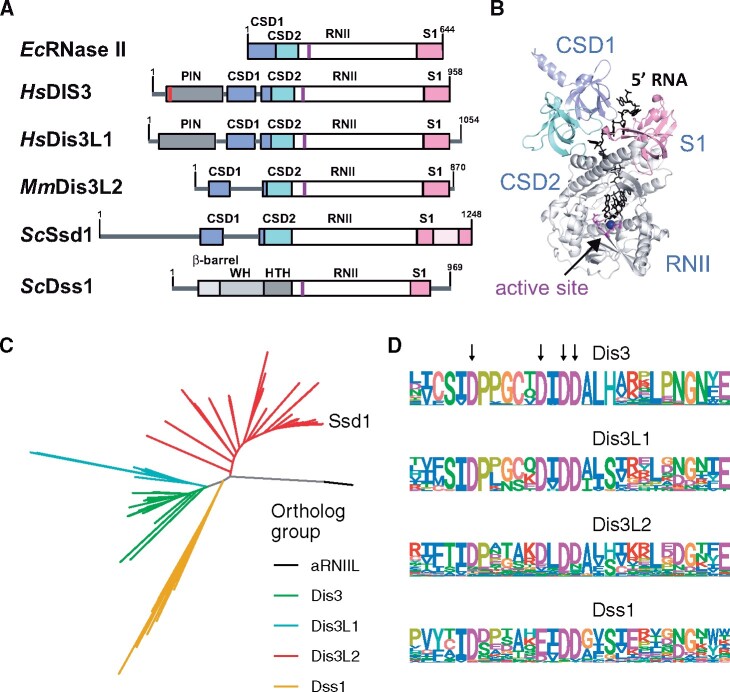
Overview of RNase II/Dis3 family enzymes and their active sites. (*A*) Domain diagrams of *Escherichia coli* RNase II and eukaryotic homologs, including human DIS3, human DIS3L1, mouse DIS3L2, budding yeast Ssd1, and budding yeast Dss1. (*B*) Key features of *E. coli* RNAse II are shown (PDB ID 2IX1) including the two cold shock domains (CSD1 and CSD2, blue and cyan), the RNase II domain (RNII, gray), and the S1 domain (pink). Substrate RNA is shown in black as sticks and the active site residues are shown in red with the Mg^2+^ ion in dark blue. (*C*) Phylogenetic tree of Dis3L2 and Ssd1 BLASTp homologs from 76 selected eukaryotes (opisthokonta and amoebozoa). Subfamilies are indicated in distinct colors: Dis3, Dis3L1, Dis3L2, Dss1, and amoebozoan RNII-Like proteins (aRNIIL). (*D*) Consensus sequences (amino acid probability) for the RNII active site in Dis3, Dis3L1, Dis3L2, and Dss1 alignments. Positions of aspartic acid residues (*D*) required for activity are marked with arrows.

RNase II family nucleases are characterized by two N-terminal β-barrel cold-shock domains (CSDs), a central funnel-shaped domain that we refer to as RNII (also called RNB), and a C-terminal β-barrel S1 domain (fig. 1*A*). The nuclease activity is conferred by a magnesium ion at the center of the RNII domain’s “funnel” (fig. 1*B*). Four conserved aspartic acid (D) residues form a motif, DxxxxxDxDD (using single amino acid code, where x is any residue), that is conserved in all known active RNase II family nucleases. The first, third and fourth D (equivalent to D201, D209, and D210 in *E. coli* RNAse II) are thought to be required for coordinating the magnesium ion (Zuo et al. 2006), whereas the second D hydrogen bonds to the 3′OH of the terminal base in the active site (Frazão et al. 2006). Mutation of these conserved aspartic acids abolished the nuclease activity of RNII domains from diverse subfamilies in prokaryotes and eukaryotes (Frazão et al. 2006; Zuo et al. 2006; Dziembowski et al. 2007; Schneider et al. 2007; Staals et al. 2010; Tomecki et al. 2010; Tomecki et al. 2010; Malecki et al. 2013; Kumakura et al. 2016). Thus, any RNase II homolog lacking some or all of these catalytic residues is likely to lack the conventional nuclease activity and may be assumed to be a pseudonuclease.

## The Ssd1 Family of Inactive RNase II-like Proteins in Ascomycete Fungi

Recent advances in fungal genome sequencing and phylogeny enable new insights into molecular evolution. The fungal kingdom is the most species-rich within the eukaryotes; the most recent common ancestor of subkingdoms dikarya and mucoromycota, over 600 Ma (Lücking et al. 2009), is likely to have lived as a multicellular filamentous organism (Kiss et al. 2019). Within dikarya, ascomycota and basidiomycota are large monophyletic subphyla that each include both multicellular filamentous and unicellular yeast species (Hawksworth and Lücking 2017; James et al. 2020). Unicellular yeasts have repeatedly evolved from filamentous ancestors, including independently in ascomycete ancestors of the model organisms *Saccharomyces cerevisiae* and *S. pombe*, and also in a basidiomycete ancestor of the pathogenic yeast *Cryptococcus neoformans* (Nagy et al. 2014).

In addition to Dis3 homologs, ascomycete yeasts contain conserved RNase II-like pseudonucleases: Ssd1 (*S. cerevisiae*; *Sc*Ssd1) lacks the conserved catalytic residues of the RNII domain and acts as an RBP (Uesono et al. 1997; Hogan et al. 2008; Jansen et al. 2009). We recently solved the structure of *Sc*Ssd1, the fold of which is conserved with Dis3 and Dis3L2 (Bayne et al. 2020). *Sc*Ssd1 was discovered due to synthetic lethality of *SSD1* alleles in combination with cell cycle mutants (Sutton et al. 1991; Wilson et al. 1991). Deletion or truncation of *Sc*Ssd1 has pleiotropic effects, including reduced tolerance of stresses arising from ethanol, heat, calcium, the kinase inhibitor caffeine, and multiple chemicals that stress the cell wall (Tsuchiya et al. 1996; Kaeberlein and Guarente 2002; Parsons et al. 2004; Mir et al. 2009; López-García et al. 2010; Avrahami-Moyal et al. 2012). Ssd1 homologs are required for virulence in diverse ascomycete fungal pathogens of humans and plants (Tanaka et al. 2007; Gank et al. 2008; Thammahong et al. 2019). Finally, *Sc*Ssd1 was recently shown to be required to support the survival of aneuploid yeast, although the mechanism remains unclear (Hose et al. 2020). Since full-length *Sc*Ssd1 binds RNA without detectable degradation, the pleiotropic effects when Ssd1 is lost presumably reflect the loss of RNA-binding, rather than nuclease activity.

Ssd1-like ascomycete pseudonucleases have conserved function and regulation. *Sc*Ssd1, and its *S. pombe* homolog Sts5 (*Sp*Sts5), were reported to act as translational repressors of specific mRNAs involved in cell growth and cytokinesis (Jansen et al. 2009; Nuñez et al. 2016). Moreover, a conserved motif was identified in the RNAs that they target (Hogan et al. 2008; Nuñez et al. 2016), indicating that the RNA-binding surface is also highly conserved. *Sc*Ssd1-mediated mRNA repression connects to networks regulating morphogenesis via the cell wall biogenesis kinase Cbk1 (Du and Novick 2002; Jorgensen et al. 2002): *Ssd1* deletion suppresses the lethality of *Cbk1* deletion. *Sc*Ssd1 is phosphorylated by Cbk1 at its N-terminus (Jansen et al. 2009). Regulation of Ssd1 by Cbk1 is conserved in diverse ascomycota, including the pathogenic yeast *Candida albicans*, the bread mould *Neurospora crassa*, and *S. pombe* (Lee et al. 2015; Nuñez et al. 2016; Herold and Yarden 2017). However, it has not been clear which are the least diverged nuclease-active homologs of *Sc*Ssd1, nor whether these homologs have overlapping function or regulation. It was also unclear whether the loss of nuclease activity in related pseudonucleases arose from a single evolutionary event or from multiple independent events.

Here we ask, how are pseudonucleases such as Ssd1 related to Dis3-family enzymes, and when did the ancestor of Ssd1 lose its nuclease activity? Our phylogenetic analysis establishes that Ssd1 is the least diverged homolog of Dis3L2 in Saccharomycete yeasts, despite its lack of an active site. We show that the active site was lost on at least four separate occasions in fungi. By contrast, the CSDs are highly conserved across both active and inactive homologs, in most branches of dikarya and mucoromycota. Based on these observations, we predicted that the nonnuclease function of Ssd1 is conserved beyond ascomycota. We verified this by demonstrating a requirement for Ssd1 in cytokinesis in polyploid “titan” cells but not euploid yeast of the basidiomycete yeast *C. neoformans*, echoing function in *S. cerevisiae* (Hose et al. 2020).

## Results, Methods, and Discussion

### Ascomycete RNase II Family Pseudonucleases Descend from Dis3L2

To understand the evolution of *Sc*Ssd1, we first checked precomputed databases of protein homology. The PANTHER protein homology database includes *Sc*Ssd1 within a single Dis3L2 phylogeny (PTHR23355:SF9, PANTHER version 15.0 [Mi et al. 2010]). The most parsimonious interpretation is that modern Ssd1 and Dis3L2 proteins are the descendants of a single eukaryotic ancestor. The OrthoDB hierarchical homology database clusters *Sc*Ssd1 with Dis3L2 and Dis3 proteins in both eukarya and fungi (groups 1104619at2759 and 67258at4751 [Kriventseva et al. 2019]). The OrthoDB group containing *Sc*Ssd1 in ascomycota excludes Dis3 (group 109571at4890), but includes some homologs in ascomycete filamentous fungi with an active site sequence signature. However, the active site has been lost in all the least diverged homologs in the saccharomycotina (OrthoDB group 8134at4891). This implies that the ancestral ascomycete had an active Dis3L2-like RNase, and that the active site was lost in the descendant of Dis3L2 in the ancestral saccharomycete.

### Reconstructing RNase II Families in Opisthokonts and Amoebozoa

To map Ssd1 and Dis3L2 evolution beyond fungi, we next performed a BLASTP search (Sayers et al. 2020) against *Sc*Ssd1, and Dis3L2 enzymes from *S. pombe* (*Sp*Dis3L2), and *Homo sapiens*, from 76 phylogenetically representative species. We focused on representative fungi with sequenced genomes including major model organisms, edibles, and pathogens, along with some animals/metazoa, other holozoa and holomycota (Torruella et al. 2015). We included amoebozoa as an outgroup. We filtered the list of BLASTP homologs to have *E*-value 1 or less, and alignment length 200aa or more, and removed truncated sequences. We then aligned the curated full-length sequences with MAFFT (Katoh and Standley 2013) and trimmed gaps at gap threshold 0.1 with trimAl (Capella-Gutiérrez et al. 2009). From the trimmed multiple sequence alignment, we created a Bayesian maximum likelihood tree using IQ-TREE 2 (Minh et al. 2020) with an LG amino acid substitution model, running on the CIPRES science gateway (Miller et al. 2015). We plotted the tree using ggtree (Yu 2020), using ggplot2 (Wickham 2016) and tidyverse packages (Wickham et al. 2019) in R markdown (Xie et al. 2018). Full data, code, and logs for these analyses are available (doi:10.5281/zenodo.3950856).

The maximum likelihood tree shows clear clusters for Dis3, Dis3L1, Dis3L2, mitochondrial homolog Dss1, and a branch of amoebozoan RNII-Like proteins (aRNIIL) that we do not pursue further (fig. 1*C*). This reproduces previous results on clustering of Dis3/Dis3L1/Dis3L2 homologs (Ustianenko et al. 2013), and is consistent with the reported domain structures of these proteins (fig. 1*A*). For example, all Dis3 homologs have an N-terminal PIN endonuclease domain with conserved catalytic residues, and Dis3L1 homologs have a PIN domain lacking essential catalytic resides, as reported for human Dis3L1 (Staals et al. 2010; Tomecki et al. 2010). Dis3 and Dis3L1 are each mostly single-copy, and Dis3L1 is found only in metazoa in both this analysis and in the PANTHER database (PTHR23355:SF35/SF30 [Mi et al. 2010]). Dss1 is absent from metazoa.

### Related RNase II Families Have Conserved Nuclease Active Site Signatures

We next computed consensus amino acid sequences for the active site of the larger clusters (fig. 1*D*), using the ggseqlogo package. This revealed a distinct active site signature for each subfamily. There is perfect conservation of the magnesium co-ordinating aspartic acids (D) in Dis3 (**D**PPgCx**D**I**DD**, where essential catalytic residues are bold, capital letters highly conserved, lower case letters indicate commonly occurring and x indicates any residue) and in Dis3L1 (**D**Pxxxx**D**I**DD**). However, both signatures for Dis3L2 (**D**Pxxxx**D**L**DD**) and Dss1 indicate that alternative residues appear in the conserved positions within this data set. This indicates that both Dis3L2 and Dss1 lineages include some family members that are probable pseudonucleases, beyond *Sc*Ssd1. Furthermore, Dss1 shows a highly conserved E residue within the active site signature (**D**xxxxx**E**L**DD**), indicating that this conservative substitution can be tolerated in some active RNase II nucleases.

### The Dis3L2 Tree Largely Matches Fungal Species Phylogenies

**Fig. 2.**
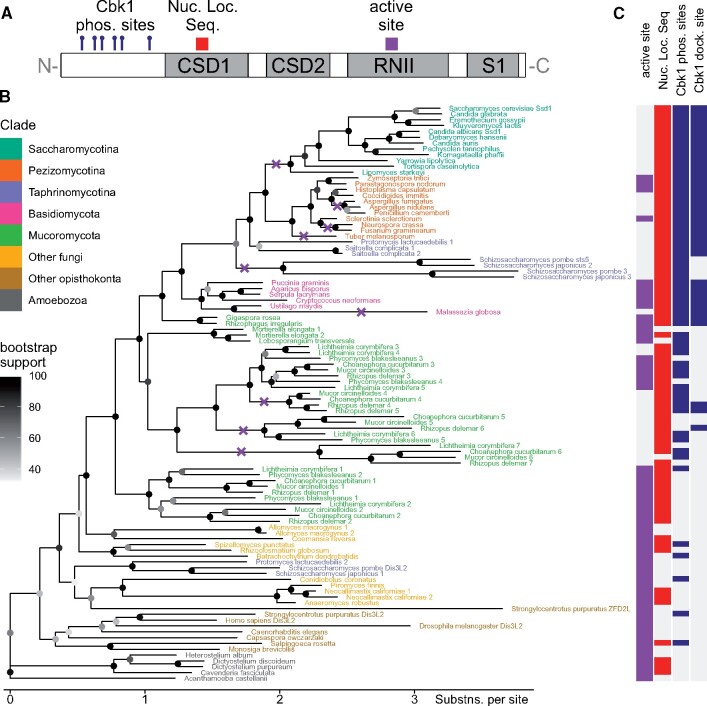
Evolution of the Dis3L2/Ssd1 family in fungi, other opisthokonta, and amoebozoa. (*A*) Schematic of features found in Dis3L2 and Ssd1 family proteins. (*B*) Phylogenetic tree of Dis3L2 family proteins, excluding N-termini aligned to *Sc*Ssd1 residues 1–337. Proteins at tips of tree are labeled by the species name colored by clade, with a further identifier where there are multiple paralogs. Note that homologs from taphrinomycotina are in widely separated groups, for example, *S. pombe* Dis3L2 and *S. pombe* Sts5. Branches containing high-confidence losses of the active site are marked with purple crosses. (*C*) Features of Dis3L2/Ssd1 family proteins shown aligned with their position in the phylogenetic tree in *B*. For example, all homologs in Saccharomycotina have no active site, a nuclear localization sequence, Cbk1 phosphorylation sites and a Cbk1 docking site. See text for details; full information with sequences, sequence identifiers, feature calculation and feature counts, in the zenodo repository online at doi:10.5281/zenodo.3950856.

We next examined the evolution of Ssd1/Dis3L2 homologs and their features (fig. 2*A*) across a focused phylogenetic tree. We first generated a new multiple sequence alignment on the Dis3L2 cluster identified above, using the more accurate local pair option in MAFFT (Katoh and Standley 2013). After removing the poorly aligned N-terminus corresponding to *Sc*Ssd1 1–337, that is predicted to be unstructured, we then computed the tree as previously (fig. 2*B*). As expected, the phylogeny of Dis3L2 homologs mostly follows species-level phylogenies as assembled from multiple genes (Ren et al. 2016). Most species in metazoa, ascomycota, basidiomycota, and chytridiomycota have one homolog each, whereas mucoromycota have multiple homologs, reflecting repeated whole-genome duplications in this clade (Corrochano et al. 2016). We did not find any Dis3L2 homologs in microsporidia or cryptomycota, which are early-diverged fungi with reduced genomes and an intracellular parasitic lifestyle (James et al. 2013). Surprisingly, homologs in taphrinomycota are placed in two widely separated groups: in *S. pombe*, *Sp*Dis3L2 seems to have diverged slowly with respect to basal opisthokonts, whereas *Sp*Sts5 clusters with other ascomycete homologs, but with a longer branch length that indicates faster sequence divergence. Repeated analyses with different gene lists and alignment parameters confirmed this wide separation (data not shown), although the exact placing of the *Sp*Dis3L2 group is poorly resolved, as indicated by the low bootstrap values.

To shed light on the evolution of Ssd1/Dis3L2 function, we next computed features of the (untrimmed) aligned protein sequences (fig. 2*A*) and displayed them alongside homologs in the tree (fig. 2*C*). The distribution of these features is not uniform across the Dis3L2 family; a detailed discussion of each follows below. An “active site signature” is identified where the three magnesium-co-ordinating Ds are in place in the RNII domain. A classical nuclear localization signal was previously characterized in a loop in CSD1 of *Sc*Ssd1 (Kurischko et al. 2011); equivalently placed conserved sequences are identified. We identified regulatory Cbk1 kinase phosphorylation sites in the N-terminal region from the consensus sequence Hxxxx[ST], including at least one positive amino acid (K or R) in the central xx residues, and the Cbk1 phosphorylation-enhancing docking site from its consensus sequence [YF]x[FP] (Gógl et al. 2015).

### The Dis3L2 Active Site Signature Is Lost in at Least Four Independent Fungal Lineages

The active site signature is present in all Dis3L2 homologs examined from amoebozoa, metazoa, and early-diverging fungi, such as chytridiomycota, indicating that the ancestral Dis3L2 was a nuclease (fig. 2*C*). The distribution of active site signatures on the phylogenetic tree indicates at least four independent losses of the active site in fungal Dis3L2 enzymes. First, the entire budding yeast saccharomycotina clade has inactive Ssd1/Dis3L2 homologs, indicating a loss of the active site in an ancestor of the entire clade. Second, filamentous fungi in the pezizomycotina have a mix of active- and inactive-signature homologs, indicating a loss of the active site in ancestors of *Aspergillus* and *Neurospora*, and potentially also independently in the ancestor of the black truffle, *Tuber melanosporum*. The active site was also lost in a protein ancestral to *Sp*Sts5-like homologs in taphrinomycota, although the exact placement of this branch is poorly resolved, so it is unclear if that was an independent event. Third, the dandruff-causing basidiomycete *Malassezia globosa* has an inactive homolog, which clusters within the active homologs of other basidiomycetes. The active site is also lost in all sequenced members of genus *Malassezia* (data not shown). Fourth, in some groups of postgenome-duplication mucoromycota homologs the active site has been lost, for example, *Rhizopus delemar* 5/6/7, despite closely related homologs with an intact active site signature, for example, *R. delemar* 3. Indeed, our phylogenetic tree shows with high confidence that the active site has been lost on multiple branches diverging from the extant active-signature *R. delemar* 3.

Most Dis3L2 homologs contain a positively charged nuclear localization sequence in a loop in CSD1, similar to *Sc*Ssd1, suggesting that nuclear localization is common in this family regardless of nuclease activity. One exception is *Sp*Dis3L2 and its active homologs in taphrinomycotina, which have lost the NLS signature in this location.

### Regulation of Dis3L2 by Kinases Is Conserved beyond Dikarya

Due to the crucial role of the cell wall biogenesis kinase Cbk1 in regulating Ssd1, we investigated the evolutionary origins of Cbk1 regulation and its co-occurrence with nuclease activity. Phosphorylation sites and docking sites recognized by Cbk1 in *Sc*Ssd1 are conserved in almost all dikarya and many mucoromycota Dis3L2 homologs (fig. 2*C*). Cbk1 phosphorylation sites are a paradigmatic example of short linear motifs that are conserved in otherwise fast-diverging disordered regions (Zarin et al. 2019). Indeed, in the otherwise poorly aligned N-terminal domain of Ssd1/Dis3L2 homologs, multiple Hxxxx[ST] phosphorylation motifs stand out as strikingly conserved, for example, seven motif instances in *S. cerevisiae*, eight instances in *C. neoformans*. A partial exception are members of the *Sp*Sts5 group that have two phosphorylation motifs but lack the Cbk1 docking site, and that are regulated by the diverged Orb6 kinase (Nuñez et al. 2016). It was previously noted that Cbk1 sites are conserved in saccharomycotina and pezizomycotina (Jansen et al. 2009). This analysis argues for even deeper conservation of Dis3L2/Ssd1 regulation, including of distant homologs with an intact active site signature.

Ssd1/Dis3L2 regulation could also involve further posttranscriptional modifications. *Saccharomyces cerevisiae* Ssd1 phosphorylation in vivo was reported to require the cyclin-dependent kinase Cdk1 (Albuquerque et al. 2008; Holt et al. 2009). However, this requirement is likely to be indirect because Cdk1 regulates Cbk1 through a signaling cascade (Mancini Lombardi et al. 2013). Measurements in cell lysates failed to detect Ssd1 as a direct Cdk1 target (Ubersax et al. 2003). We did not pursue Cdk1 regulation further here because the two Cdk1 consensus sites [S/T]Px[K/R] on *Sc*Ssd1 are not conserved in our alignment, and the Cdk1-dependent sites indirectly identified in vivo overlap with verified and conserved Cbk1 phosphorylation sites.

### Ssd1 CSDs Are Highly Conserved in Dikarya and Mucoromycota, Compared with RNII Domains

**Fig. 3.**
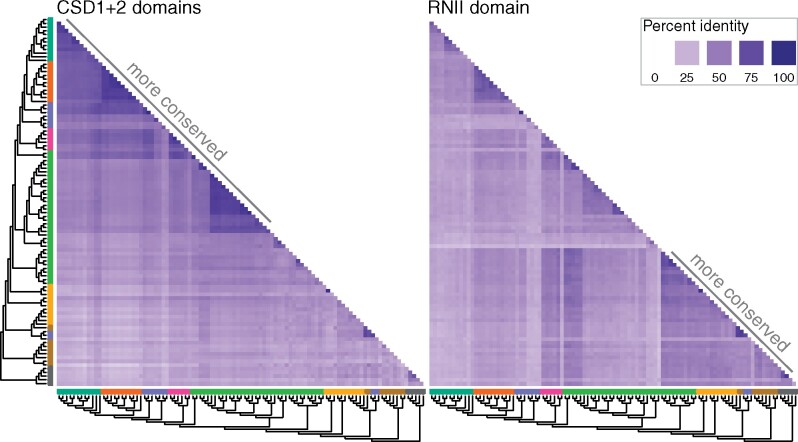
Conservation of Dis3L2-family domains in fungi, other opisthokonta, and amoebozoa. Heatmap shows percent identity of alignments within specific domains CSD1 and CSD2 considered together, and RNII domain, with darker blues indicating higher conservation. For example, dark blue patches at top left of CSD1 + 2 indicate that these domains are highly conserved within dikarya, compared with the lighter colors in the corresponding region for RNII indicating lower conservation. By contrast, dark blue patches at the bottom right of RNII indicate that this domain is highly conserved in early-diverging fungi, other opisthokonts, and amoebozoa, compared with CSDs. Cladogram and clade coloring is repeated from figure 2, as these are calculated from the same sequences in the same order.

We next examined the sequence conservation patterns of Ssd1/Dis3L2 (fig. 3), which indicate differential conservation of function in distinct domains. We computed pairwise percent amino acid identity in the trimmed MAFFT alignments for CSDs 1 & 2 (*Sc*Ssd1 338–659) and the RNII domain (*Sc*Ssd1 689–1014), shown in figure 3 in the same sequence order as the tree in figure 2*B*. The CSDs are highly conserved within dikarya, especially within close homologs of *Sc*Ssd1 in saccharomycete yeast. The CSDs remain highly conserved in ascomycete and basidiomycete Ssd1/Dis3L2 homologs that retain an active site sequence signature, and in some homologs in mucoromycota. CSDs are much less well conserved in early-diverging fungi, metazoa and amoebozoa, contrasting with the higher conservation of RNII domains in these Dis3L2 nucleases. By contrast, the RNII domains are less well-conserved than CSDs in ascomycetes. However, the RNII domains are well-conserved within active-signature nucleases in the basidiomycota, with the exception of pseudonucleases in *Malassezia*. These conservation patterns suggest a new function emerging in the CSDs in a nuclease-active ancestor of Ssd1 in dikarya and mucoromycota. Subsequently, the CSDs were highly conserved, whereas RNII domains were less conserved and repeatedly lost nuclease activity.

Our results may explain why previous reports focusing on nuclease activity in the RNII domain have argued that *S. cerevisiae* lacks a Dis3L2 homolog (Lubas et al. 2013; Malecki et al. 2013), as the active site region of the RNII domain is particularly diverged. By contrast, phylogenetic analysis and conservation of the CSDs place Ssd1 unambiguously as the least-diverged homolog of Dis3L2 in Saccharomycotina.

### Ssd1 Has a Conserved Role in Cytokinesis in the Basidiomycete Yeast *C. neoformans*

**Fig. 4.**
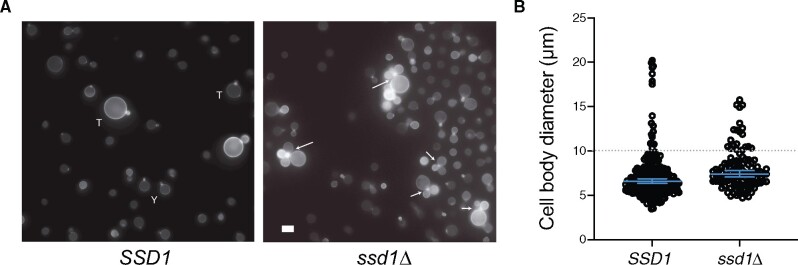
*Cryptococcus neoformans* Ssd1 is required for cytokinesis from polyploid titan-phase growth but not yeast-phase growth. *SSD1* (wild-type strain H99) and *ssd1Δ C. neoformans* were grown in titan-inducing conditions as previously described (Dambuza et al. 2018). (*A*) Cells were stained for chitin using 0.1 μg/ml calcofluor white and imaged using a Zeiss AxioImager at 63×. Scale bar indicates 10 μm. Y indicates representative yeast cells, T indicates representative titan cells, and arrows indicate cells with abnormal cytokinesis. Among WT mother cells, none were observed with more than one bud; among *ssd1Δ* cells, the majority of mother cells >10 μm had two or more daughters associated with the bud neck. (*B*) The diameter of >100 cells was measured and analyzed by Mann–Whitney *U* test for nonparametric data (*P* < 0.0001). Median diameter and 95% CI are shown. All cells in five randomly selected frames were measured. Data are representative of three independent repeats but only a single experimental repeat is shown.

To examine conservation of function associated with conservation of features between ascomycota and basidiomycota, we analyzed the Ssd1/Dis3L2 homolog in the basidiomycete yeast *C. neoformans*. *Cn*Ssd1 is of interest because it retains a nuclease active site signature but also has features related to inactive Ssd1 homologs (nuclear localization signal, Cbk1 docking and phosphorylation sites). We used the *ssd1Δ*/CNAG_03345 ORF deletion from the Madhani laboratory deletion collection in the H99 background (Chun and Madhani 2010). Previous analysis of *ssd1Δ* found a slight growth defect, but no impact on yeast-phase morphogenesis (Gerik et al. 2005); we were able to replicate these findings during yeast phase growth in rich medium (data not shown). *Cryptococcus neoformans* display two different morphologies: A haploid yeast-phase budding morphology and a much larger, polyploid “titan” morphology (>10 μm), that is associated with aneuploidy and virulence (Zaragoza and Nielsen 2013; Zhou and Ballou 2018). In vitro titan induction of wild type cells (*SSD1*) yields a mixed population of both yeast-phase and titan cells (fig. 4*A*) (Dambuza et al. 2018). Under this condition, the *ssd1Δ* strain shows defects in cytokinesis specifically in titan cells (fig. 4*A*). Among *ssd1Δ* cells, the average cell diameter was roughly 2 μm greater than *SSD1* cells, and the majority of mother cells >10 μm had two or more daughters associated with the bud neck (*P* < 0.0001, Mann–Whitney *U* test; fig. 4*B*). We observed no morphological or growth defects in yeast-phase cells that are also present during titan induction (fig. 4*A*).

In contrast to previous reports of *Cn*Ssd1 playing no role in morphogenesis, our observation suggests a conserved role for *Cn*Ssd1 in cytokinesis. However, this role is either specialized to polyploid titan morphology or is redundant with other regulators during yeast-phase growth. These findings are consistent with those of Hose et al. showing that loss of *Sc*Ssd1 function is lethal for aneuploid cells but not euploid cells (Hose et al. 2020). Overall, the data suggest that these conserved functions are not related to nuclease activity, lacking in *Sc*Ssd1, but may instead be connected to Cbk1 regulation. The Cbk1 kinase is required for cytokinesis from yeast-phase growth in *C. neoformans* (Walton et al. 2006), and we speculate that this reflects Cbk1-mediated regulation of RNA binding by Ssd1.

### Evolution of an Inactive RBP from an Ancestral Nuclease via a Bifunctional Intermediate

Our work suggests a scenario where an ancestral Dis3L2 nuclease evolved a second RNA-binding function in a common ancestor of dikarya and mucoromycota. This ancestral fungus was likely developing a multicellular lifestyle involving spatially extended hyphal growth (Kiss et al. 2019). Given the reported role of modern-day Ssd1 homologs in mRNA localization and translational control, we speculate that this role was played by Ssd1 in the ancestral hyphal fungus.

Nucleases can display weak RNA-binding activity on surfaces distal to the active site, as a means of increasing their affinity for substrates. These additional sites can be adapted during evolution, leading to bifunctionality in these enzymes, which can be followed by loss of the nuclease activity. Our results show multiple independent losses of nuclease activity in fungal homologs of Dis3L2, subsequent to the emergence of a conserved sequence pattern in the CSDs. We speculate that this conserved sequence pattern reflects the evolution of a novel RNA-binding function in the CSDs. The opisthokont exosome provides a more extreme example of pseudonuclease evolution: Here, six core PH nuclease-like proteins have all lost activity compared with their homologs in the archaeal exosome and bacterial PNPase (Houseley and Tollervey 2009). Like *Sc*Ssd1, these core proteins are pseudonucleases with a role in RNA binding. Nuclease activity in the opisthokont exosome is now restricted to the Dis3 subunit, or to the homologous Dis3L1 subunit of the metazoan cytoplasmic exosome (Staals et al. 2010; Tomecki et al. 2010). Even there, the PIN domain of Dis3 is an active endonuclease, yet the PIN domain of Dis3L1 is a “pseudonuclease domain” that lacks nuclease activity whereas ensuring Dis3L1 binds to the core exosome. Previously reported pseudonucleases in animals include EXD1 (Yang et al. 2016), SMG5 (Glavan et al. 2006), *Maelstrom* (Chen et al. 2015), and *Exuperantia* (Lazzaretti et al. 2016). Thus, pseudonucleases are a common feature of complexes that bind and regulate RNA.

Although the evidence is unambiguous that RNII domains lacking catalytic D residues are inactive, proteins that retain these residues are not necessarily active, because nuclease activity could be blocked by other means. For example, access to the active site may be blocked by mutations that occlude the RNA-binding channel. Indeed, our structure of *Sc*Ssd1 indicates that access to the former active site is blocked by loop insertions in multiple domains (Bayne et al. 2020). However, the strong conservation of the active site and RNII domain in some clades, such as most basidiomycota, argues that retained nuclease activity is likely in these clades. Future experiments will have to address if diverged Dis3L2 homologs, such as *Cn*Ssd1, are active nucleases in vivo or in vitro, and if the nuclease activity is required for wild-type cell growth.

Lastly, we note that the canonical nuclease function of Dis3L2 enzymes requires their canonical substrates: RNAs that have poly(U) tails added by terminal U-transferases such as *S. pombe* cid1 and cid16, and human TUT1, TUT4, and TUT7 (Yashiro and Tomita 2018). In the absence of terminal U-transferase activity, there would be few poly(U)-tailed substrates, removing selective pressure to retain Dis3L2’s terminal U-targeted nuclease activity. In this context, a bifunctional RNase/RBP would be unconstrained to evolve into a monofunctional RNA-binding pseudonuclease. Conversely, if terminal U-targeted nuclease activity were lost, there might be pressure against retaining an active TUTase, to avoid accumulation of poly(U)-tailed substrates. Supporting the coevolution of Dis3L2 and TUTase enzymes, TUTases homologous to *Sp*cid1/cid16 are present in fungal clades with active-signature Dis3L2 such as taphrinomycotina, most basidiomycota, mucoromycota, and chytridiomycota (PANTHER: PTHR12271: SF40; OrthoDB: 264968at4751), but absent from prominent clades lacking active Dis3L2, such as most saccharomycotina.

Overall, our analysis identifies extant fungal Ssd1 pseudonucleases as descendants of the Dis3L2 family of 3′–5′ exoribonucleases, identifies the CSDs as highly conserved features across dikarya that are likely to perform conserved functions related to aneuploidy and cytokinesis, and raises new questions about the interaction of these domains with client RNAs.
